# Supporting international networks through platforms for standardised data collection—the European Registries for Rare Endocrine Conditions (EuRRECa) model

**DOI:** 10.1007/s12020-021-02617-0

**Published:** 2021-01-29

**Authors:** S. R. Ali, J. Bryce, C. Smythe, M. Hytiris, A. L. Priego, N. M. Appelman-Dijkstra, S. F. Ahmed

**Affiliations:** 1 Developmental Endocrinology Research Group, Royal Hospital for Children, University of Glasgow, Glasgow, UK; 2 Office for Rare Conditions, University of Glasgow, Glasgow, UK; 3 Department of Medicine, Division of Endocrinology, Leiden University Medical Center, Leiden, The Netherlands

**Keywords:** Registries, Databases, European Reference Networks, Endocrinology, Rare diseases, Rare conditions

## Abstract

Rare endocrine pathology is manifested by either a deficiency or excess of one or more hormones. These conditions can be life-threatening and are almost universally associated with long-term morbidity. Understanding the aetiology of these conditions requires multicentre collaboration and expertise, most often across national boundaries, with the capacity for long-term follow-up. The EuRRECa (European Registries for Rare Endocrine Conditions) project (www.eurreca.net), funded by the EU Health Programme, aims to support the needs of the wider endocrine community by maximising the opportunity for collaboration between patients, health care professionals and researchers across Europe and beyond. At the heart of the EuRRECa collaboration is a Core Endocrine Registry that collects a core dataset for all rare endocrine conditions that are covered within Endo-ERN. The registry incorporates patient reported markers of clinical outcome and will signpost participants to high-quality, disease-specific registries. Furthermore, an electronic surveillance programme (e-REC) captures clinical activity and epidemiology for these rare conditions. EuRRECa receives guidance compliant with the highest ethical standards from Expert Working Groups that align with the Main Thematic Groups of Endo-ERN. Security, data quality and data governance are cornerstones of this platform. Clear policies that are acceptable to patients, researchers and industry for data governance coupled with widespread dissemination and knowledge exchange through closely affiliated stakeholders will ensure sustainability beyond the current lifetime of the project. This paper describes the infrastructure that has been developed, stakeholder involvement, the data fields that are captured within the registry and details on the process for using the platform.

## Background

Endocrine conditions are often associated with chronic long-term morbidity and optimal management of these conditions requires specific expertise and the capacity for long-term follow-up in expert centres providing multidisciplinary care. Lack of expert and evidence-based care, particularly in rare conditions, can result in substantial variation in patient care. Registries have the potential to improve patient care by enabling pooling of data and facilitating surveillance, audit and research via a virtual environment [1]. The importance of international registration of patients with rare conditions is widely recognised and supported by the European Union [2], with several European initiatives including Orphanet and RD-Connect highlighting existing registries [3, 4].

The recent development of the European Reference Network for Rare Endocrine Conditions (Endo-ERN) (endo-ern.eu), has provided an opportunity to consider how to pool resources and develop a virtual environment that serves the objectives of this ERN and acts as a model for the wider endocrine community and beyond. Endo-ERN is the largest ERN with 71 Reference Centres from 19 member states that are estimated to care for over 60,000 patients. Endo-ERN includes 36 groups of conditions with orphacodes that are organised into eight ‘Main Thematic Groups’ [5]. The European Commission states that ERNs require established frameworks for the gathering of standardised information on established outcomes, process indicators and patient registries [6]. Endo-ERN is able to act as a model for a large group of rare conditions that require a common Core Endocrine Registry (CER) and an electronic surveillance system to capture activity, epidemiology and natural history of these conditions.

## The concept of EuRRECa

**Fig. 1:**
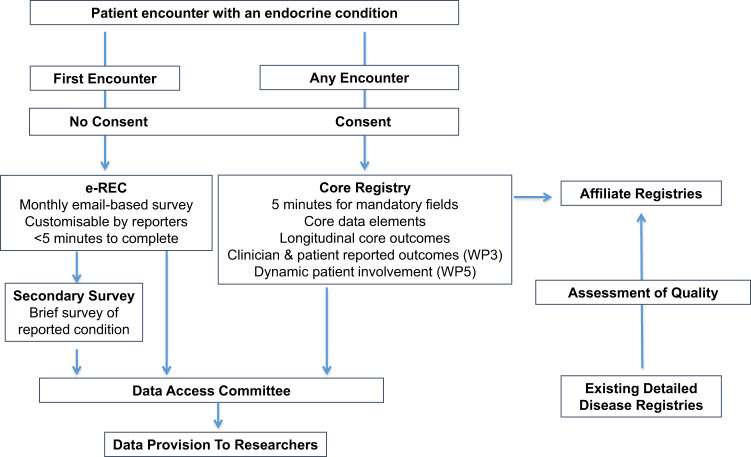
The concept of the European Registry for Rare Endocrine Conditions (EuRRECa)

EuRRECa (https://eurreca.net/) was launched in February 2018 and is funded by the EU Health Programme and is also supported by the European Society for Paediatric Endocrinology (ESPE) and the European Society of Endocrinology (ESE). It aims to maximise the opportunity for patients, health care professionals and researchers to participate and use high‐quality, patient-centred registries for rare endocrine conditions that are covered within Endo‐ERN. Participation is open to all members of Endo-ERN and to all other professionals providing endocrine care. EuRRECa works closely with Endo-ERN, ESPE and ESE and the platforms it has developed have most recently been adopted by the European Reference Network for Rare Bone Conditions (ERN-BOND) and its related registry, the European Registry for Rare Bone and Mineral Conditions (EuRR-Bone). The two main arms of the EuRRECa project encompass—a CER that collects a common dataset and clinician and patient reported outcomes and an electronic surveillance system, the e-Reporting for Rare Endocrine Conditions (e-REC) programme (Fig. 1).

**Fig. 2:**
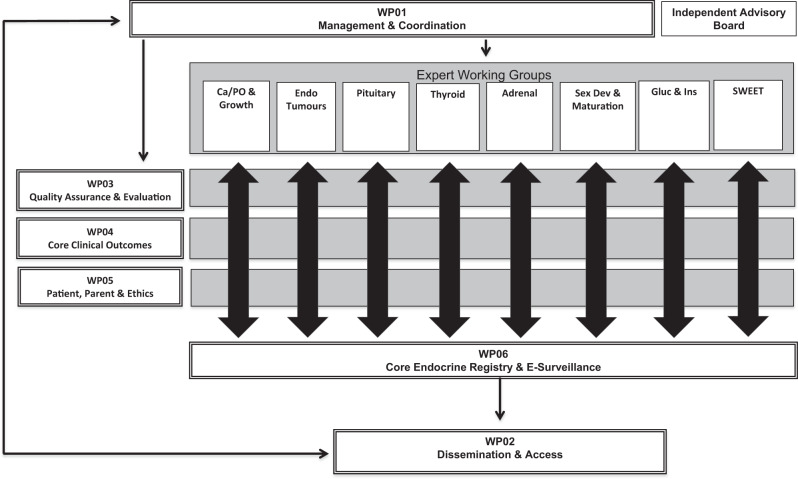
The European Registry for Rare Endocrine Conditions (EuRRECa) consortium. WP01-06: Work Packages 01–06

EuRRECa receives guidance from Expert Working Groups that align with the Main Thematic Groups of Endo-ERN. This guidance flows through work packages (WP) that review the needs of patients and parents, comply with the highest ethical standards, evaluate the quality and interoperability of datasets and combine them with patient-centred outcomes (Fig. 2). Clear policies that are acceptable to patients, researchers and industry for data governance coupled with dissemination and knowledge exchange through closely affiliated stakeholders will ensure that EuRRECa is sustained beyond the current lifetime of the project.

## Stakeholders involved in the development of EuRRECa

Stakeholders beyond the project group include patient and parent groups, government organisations, European reference networks, national and European initiatives including Orphanet and industry and pharma. Until now, in the field of endocrine conditions, interaction between professional societies, the pharmaceutical industry and registries held in academic centres has been limited. EuRRECa has developed standard policies that provide equitable access and have a system of quality assurance that fulfil the need of industry and regulators. The involvement of patients and parents of children with rare conditions in registries is also vital and patient organisations such as EURORDIS appreciate the need for internationally available, interoperable, high quality registries that have a ‘patient-centred’ approach. The framework structure of EuRRECa ensures comprehensive patient representation in all of its working groups as well as its project governing board and independent governing board (Fig. 2). In addition, there is a work package dedicated for the needs of patients and parents, with individuals from a multi-lingual and multi-disease background. Professional societies such as ESPE and ESE widely support EuRRECa and provide a valuable forum for exchanging and disseminating information. Moreover, close collaboration between adult and paediatric endocrinologists has been evident through previous registry-based European projects including EuroDSD, DSDnet, DSDlife and EU TAIN (www.tain-project.org). Going forward, utilising the model and resources that professional endocrine societies in Europe already have in place for supporting registries for rare endocrine conditions will be instrumental in ensuring the long-term sustainability and capacity building for the EuRRECa project.

## The platform for e-reporting of rare endocrine conditions (e-REC)

Many clinical and scientific networks for rare conditions operate an electronic reporting system to capture activity as well as to understand incidence, prevalence and natural history of conditions, whilst supporting research [7, 8]. e-REC (https://eurreca.net/e-rec/) is an electronic reporting system which allows continuous reporting of core indicators of activity and enables clinical networks such as Endo-ERN to embark on its stated mission of objectively mapping conditions and related activity, providing a better understanding of the occurrence of the rare conditions covered within Endo-ERN [5]. More recently, this list of conditions has been extended to include those that are covered within ERN-BOND. The platform was originally launched in 2018 and is open to all centres that look after people with such conditions. The potential for e-REC to act as an agile and adaptable tool for surveillance has also been illustrated by its use since April 2020 on a joint project with the ESE’s RD Committee for reporting new cases of COVID-19 infection in those with an existing endocrine or metabolic condition.

In the current e-REC platform, users are asked to report any new case of any of the conditions that have been included in Endo-ERN, this process does not require informed consent from patients. On reporting a case, no personally identifiable information is collected and the form itself takes <2 min for completion. The platform offers several user benefits and these include a self-registration link for new users, direct log-in to the website to view dashboards and users are also able to select conditions and age groups that they would like to report on. Multiple reporters can be selected within each reporting centre, however, it is not possible for another team member to sign up for the same condition/age group. The reporting months remain open for a period of 3 months and users have the option to report suspected and/or confirmed cases. The latter is particularly useful in cases where genetic or biochemical diagnostic confirmation is pending. Unique IDs for reported cases are generated instantaneously and emailed to users to be stored locally at reporting centres, linking cases to the unique ID will enable clarification of diagnostic certainty at a later stage. Users are able to view their reported cases and results in their dashboard with the option of downloading data in a CSV file format. The reported data are stored on a secure server in the University of Glasgow. This project complies with EU GDPR [9] and is approved by the Information Governance authorities at the NHS Greater Glasgow & Clyde Health Board and the National Research Ethics Service in the UK.

The e-REC platform is openly publicised so that all stakeholders including patients are fully aware of the reporting process and information sheets including a public information sheet are available in more than ten languages. Data are available to all stakeholders following approval by the Data Access Committee of the EuRRECa project.

## The Core Registry

The EuRRECa Core Registry (https://eurreca.net/core-registry/) has been operational since June 2019 and it collects a core dataset that has been evaluated against existing FAIR standards [10]. The fields have a high level of interoperability for a wide range of rare conditions including those that are covered within Endo‐ERN and ERN-BOND [5]. The fields that are used to collect core information include the core data elements that are recommended by European standards for data collection [11] and a high proportion of them have universal identifiers. Details of the standard operating procedures are available at the EuRRECa project website (eurreca.net/data-access-process/). Clinicians can enter patient data into the Core Registry after obtaining consent from patients. Reference centres within Endo-ERN may consider using the consent form provided by the ERN, however, more detailed information sheets and consent forms that have ethics and information governance approval have been created by EuRRECa and are available in several languages. Those centres that recruit through a process of opt-out consent can specify this information when a new record is created irrespective of the nature of the consent process, participating centres and clinicians are advised to seek local information governance and ethics approval. Ethical approval is also in place to facilitate the exchange of data in the CER and other EuRRECa-approved registries. The data collected in the registry will be used to improve clinical care as well as research with data access governed by a Data Access Committee. The results of any studies performed will be disseminated widely and the registry advises participants on other suitable studies.

Patients are able to access the registry to view their own record, set preferences for data sharing and complete patient reported outcomes (PROs); patients are required to provide their email address to their clinician for online access and this is captured on the registry consent form. PROs are increasingly being used in registries to understand patient experiences and preferences. As highlighted at the First International Rare Diseases Research Consortium (IRDiRC) Task Force Workshop on patient-centred outcome measures (PCOMs), in rare disease research as well as clinical care, quality of life data and PROs are particularly important when well-defined, widely accepted clinical outcomes are not available and a number of validated instruments already exist [12, 13]. Within the CER, generic PROs are completed using the age-specific EQ-5D tool (euroqol.org) and both clinicians and parents can use the tool to report outcomes. Work is also ongoing amongst the EuRRECa Expert Working Groups to identify condition-specific PCOMs that may be a combination of PROs and other measures observed clinically or via biomarkers. Standardising the development of PCOMs through a dedicated work package and incorporating PROs into our platform will ensure that the collection of PCOMs becomes routine enabling Endo-ERN to achieve its objective of improving patient-centred care for those with rare endocrine conditions.

## Data access, data quality and data governance

The EuRRECa project aims to promote good standards of practice by adherence to the highest standards of data security and the data collected in e-REC and the CER are subject to stringent governance [14]. The project complies with the UK Data Protection Act (2018) and General Data Protection Regulation (GDPR 2016/679) and the e-REC and the Core Registry have been approved by the UK Research Ethics Service. In addition, all participating centres obtain their own local institutional approvals for using the two registries.

The Data Access Committee (DAC) is composed of members of the Project Governing Board responsible for co-ordination, management, quality assurance, core outcomes, patients, parents and ethics and a statistician. There is also representation from ESPE and ESE. Members of the expert working groups are co-opted depending on the endocrine theme. The Committee has developed a Data Access Policy which oversees the process for requesting access to the data that are collected in e-REC and the CER [14]. Stakeholders request data by completing a Data Request Form and a Data Sharing Agreement. The request is reviewed by the DAC who provide feedback to the applicant, who may be asked to revise the request. Once approved, data are released to the applicant. In some cases, further information may be required and via the project management team, they can ask for additional data or clarification of data they have already received. This process will lead to an improvement in the quality of the data held in the registry. In addition, EuRRECa has a work package that oversees Quality Assurance. This work package was instrumental in ensuring the data being collected in the CER adhere to FAIR principles. By using Orphacodes and LOINC codes, the data are highly interoperable with other registries that use the same approach. Data may be requested for a number of reasons that may include conducting an epidemiological survey for a particular condition, designing an additional questionnaire, appraising best clinical practice or comparing outcome measures across different conditions or centres. In all cases, supply of data is dependent on feedback from the recipients in the form of a report which is posted on the public area of the website.

## Summary and future direction

The EuRRECa project has incorporated the development of a CER and an e-Reporting Programme for Rare Endocrine Conditions, with adherence to the highest standards of data security and information governance. These secure web-based platforms facilitate the collection of standardised data internationally, enabling multicentre collaborative research to be performed. Work on this project is ongoing with active developments from a clinical and research prospective, including signposting Core Registry users to Endo-ERN accredited disease registries. The lessons learnt from this model can be applied to any large group of rare conditions that require a common Core Registry and an electronic surveillance system to capture activity, epidemiology and natural history of these conditions.
